# Effect of simethicone on the bactericidal efficacy of a high-level disinfectant

**DOI:** 10.1099/jmm.0.001902

**Published:** 2024-10-04

**Authors:** Gregory G. Anderson, Katharine Segars, Anastacia M. Sanchez, Jon W. Weeks, Shanil P. Haugen, Ruchi Pandey

**Affiliations:** 1 Division of Biology, Chemistry, and Materials Science, Office of Science and Engineering Laboratories, Center for Devices and Radiological Health, US Food and Drug Administration, Silver Spring, MD 20993, USA; 2 Office of Product Evaluation and Quality, Center for Devices and Radiological Health, US Food and Drug Administration, Silver Spring, MD 20993, USA

**Keywords:** disinfectant, medical device, microbial soil, reprocessing, simethicone

## Abstract

**Introduction.** Simethicone is an over-the-counter product that is frequently used by clinicians during endoscopic procedures to reduce foaming and improve visualization. Published studies have found simethicone residue on endoscopes after cleaning and disinfecting the devices as per the manufacturer’s instructions. Some literature suggests that simethicone residue may reduce disinfection efficacy and increase the risk of patient infections.

**Gap Statement.** However, there appears to be a lack of direct evidence in the literature to either disprove this or correlate simethicone presence with an increased microbial risk.

**Aim**: Research was conducted to evaluate the *in vitro* impact of simethicone on disinfection efficacy.

**Methodology.** Bacteria were grown in a microtitre plate assay in the presence of a range of simethicone concentrations and then treated with a disinfectant. Bacterial growth was assessed by spotting each microtitre well onto an agar plate.

**Results.** The results demonstrated that, under the conditions tested, simethicone did not reduce the efficacy of Cidex ortho-phthalaldehyde disinfectant, which demonstrated at least a 6-log unit reduction in bacterial viability. Additional experiments showed that direct exposure to 66 mg ml^−1^ of simethicone reduced bacterial viability.

**Conclusion.** These results indicate that simethicone may not reduce the bactericidal efficacy of disinfectant during reprocessing, under certain conditions.

## Introduction

One of the major concerns with reusable medical devices is the risk of infection caused by the transfer of microbes from one patient to another. Patient soils may harbour medically relevant microorganisms that can attach to or otherwise coat a device, such as an endoscope [1,4]. As per US Code of Federal Regulations Title 21 Part 820 (21 CFR 820), reusable medical devices must have validated instructions for use demonstrating the device can be reprocessed [5], and in clinical settings, these devices must undergo reprocessing between patients. In the USA, reprocessing commonly includes cleaning followed by either low-level disinfection, intermediate-level disinfection, high-level disinfection (HLD) or sterilization [5,7]. Reprocessing steps are determined by the manufacturer through consideration of the Spaulding Classification [5,8, 9], depending on the level of patient contact and intended use of the device. Improper or incomplete reprocessing can dramatically increase the risk of infection by failing to adequately remove, inactivate and/or kill associated microbes.

During gastrointestinal endoscopic procedures, simethicone solutions have been incorporated as a visualization aid by reducing foam that may be generated in the gastrointestinal tract [10]. Simethicone applications include administration through endoscope channels or ingestion by mouth prior to the procedure [10]. Several studies have looked for simethicone remaining in endoscope channels after clinical use. These studies have employed methods such as borescope imaging and Fourier transform infrared spectroscopy to determine the presence of simethicone; additionally, assays to quantify ATP, protein and haemoglobin were used as indicators for residual soil masked by simethicone during reprocessing [11,13]. These studies indicated that simethicone residuals may remain within the channels of clinically used endoscopes after reprocessing, and questions have been raised as to the impact this may have on reprocessing and HLD efficacy [11,13]. In 2019, the Gastroenterological Society of Australia issued a position statement recommending

1. ‘Continued use of simethicone’ due to its benefits for visualization;
2. Using ‘the smallest effective quantity’ (e.g. 0.024–0.036% w/v);
3. Administering simethicone ‘orally or through any endoscope irrigating channel’;
4. ‘Strict adherence to instrument reprocessing protocols’, including prompt decontamination after endoscope use [14].

Additionally, with approval by the American Society for Gastrointestinal Endoscopy, a consortium of medical professional societies recommended, in 2021, using the lowest concentration (≤0.5%) and volume of simethicone and administering it through the endoscope working channel [15]. However, it is generally agreed that further studies are needed to understand appropriate simethicone concentrations to be used and the impact that residual simethicone may have on the efficacy of HLD [11].

This research aims to provide an *in vitro* evaluation of whether simethicone decreases the bactericidal efficacy of a selected disinfectant, ortho-phthalaldehyde (OPA). The effects were tested on four clinically relevant bacteria that have been known to contaminate endoscopes: *Escherichia coli*, *Pseudomonas aeruginosa*, *Klebsiella pneumoniae* and *Staphylococcus aureus* [4,16,18]. It was found that simethicone solutions did not affect bactericidal efficacy against the four medically relevant bacteria at 10^6^–10^7^ c.f.u., under the conditions tested. While it is important to note that endoscopes need to undergo full cleaning to remove gross soils prior to subsequent reprocessing steps, the results presented here suggest that residual simethicone might not impede the efficacy of the disinfectant, OPA, under the conditions tested.

## Methods

### Bacterial strains

The test organisms evaluated were *P. aeruginosa* (ATCC 15442), *E. coli* (ATCC 35378), *K. pneumoniae* (ATCC BAA1705) and *S. aureus* (ATCC 6538). All strains were grown in tryptic soy broth (TSB).

### Simethicone microtitre assay

Bacterial strains were grown in a shaking culture at 37 °C overnight in TSB. These were then diluted in TSB to an OD at 600 nm of 0.9–1.1 to achieve a final concentration of 10^9^ c.f.u. ml^−1^. Preliminary testing indicated that each strain achieved 10^9^ c.f.u. ml^−1^ at this OD (not shown). This initial inoculum was further diluted to 10^6^ c.f.u. per well for experiments, as described later. Bacterial concentration was verified by serial dilution and plating on tryptic soy agar (TSA) plates.

The microtitre assay tested the simethicone concentrations of 66, 33, 16 and 8.2 mg ml^−1^. These concentrations were achieved by adding 198 µl of Mylicon Infant Gas Relief Drops for Infants and Babies, Dye Free (Amazon B011EVP6WA) to each of the 12 wells of a 96-well microtitre plate (Falcon 351172). This commercially available simethicone formulation is supplied at a concentration of 66 mg ml^−1^; thus, 66 mg ml^−1^ simethicone was the highest achievable concentration in the test assays. Next, using a multichannel pipettor, 99 µl of the simethicone from these initial wells was serially diluted (1 : 1) into TSB across three adjacent wells of the microtitre plate, removing and discarding 99 µl from the last set of wells so that the volume in each well was 99 µl. Wells were mixed 5–10 times between dilutions. One microlitre of the prepared 10^9^ c.f.u. ml^−1^ bacteria was then added to each well, resulting in a final level of 10^6^ c.f.u. per well. Thus, the assay comprised four sets of wells of each simethicone concentration for each of the three time points. Plates were then incubated statically at 37 °C for 4±2, 18±2 and 24±2 h, as indicated in each table. At each time point, two sets of wells of each simethicone concentration (66, 33, 16 and 8.2 mg ml^−1^) received 50 µl disinfectant (Cidex OPA, Advanced Sterilization Products 20390), and the other two sets of wells of each simethicone concentration received 50 µl PBS in lieu of disinfectant. The plate was then incubated for 15 min at room temperature. While the disinfectant manufacturer’s instructions for use indicate a 12-min exposure of a device to the OPA solution, the logistics of this study necessitated the addition of disinfection into the bacterial culture present. Due to this disinfectant dilution, the exposure time was correspondingly increased to 15 min. Subsequently, one set of wells with disinfectant and one set of wells without disinfectant then received 50 µl of Dey/Engley (D/E) neutralizing broth (BD Difco DF0819-17-2) to neutralize the disinfectant, and the other two sets of wells received 50 µl PBS. Plates were then incubated for 12 min at room temperature. Following the incubation time, 10 µl from each tested well was spotted onto a TSA plate, and the plate was incubated at 37 °C overnight. In this manner, growth was tested in each of the four simethicone concentrations at three time points, each treated in four different ways: disinfectant and neutralizing broth, disinfectant alone, neutralizing broth alone and PBS (no disinfectant or neutralizing broth). Controls were inoculated with 1 µl of the bacterial culture into 99 µl TSB (no simethicone) in 12 additional wells: three wells for TBS-only growth control, three wells for control for the disinfectant-treated samples, three wells for control for the neutralizing broth-treated samples and three wells for control for the samples treated with disinfectant and neutralizer.

**Fig. 1.**
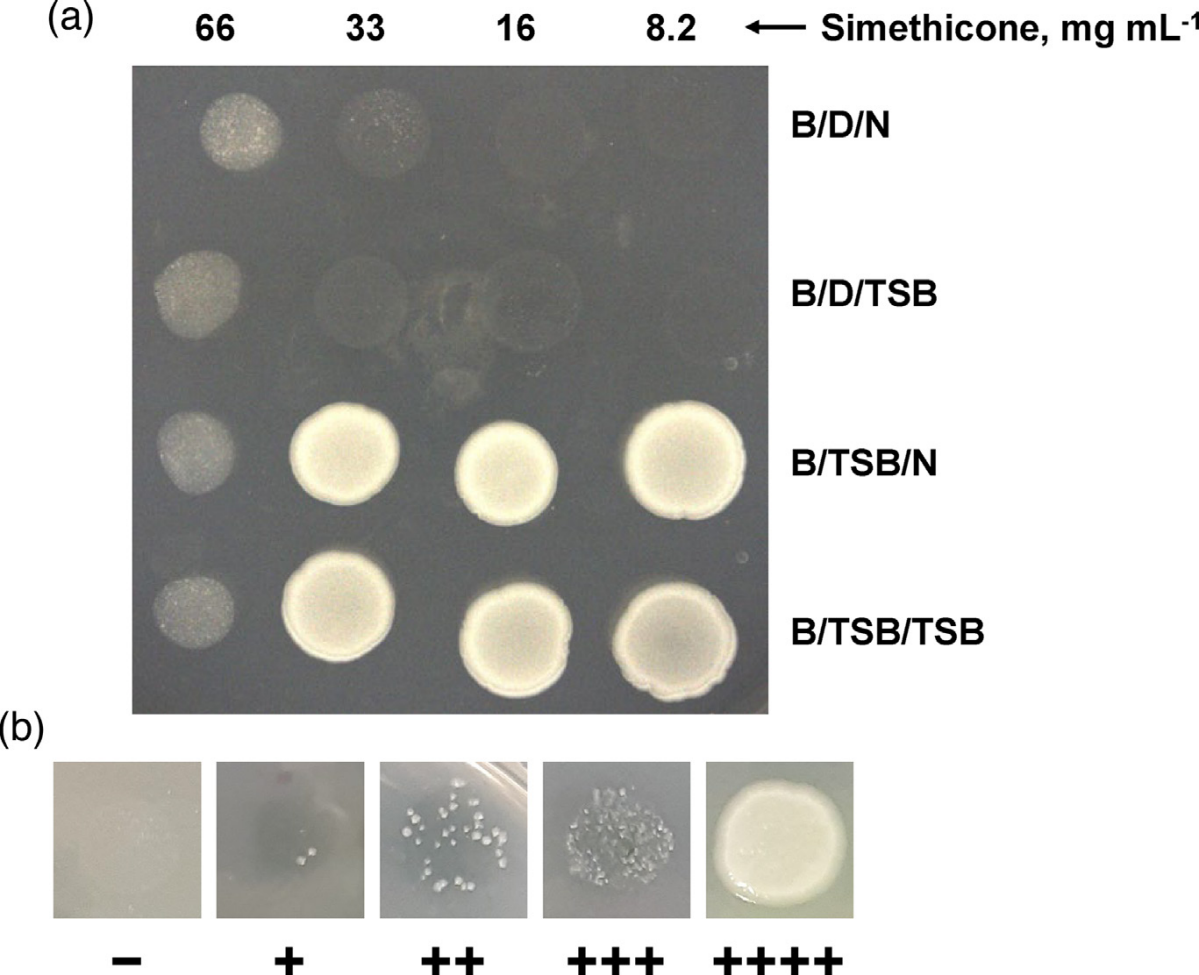
Example spots of the test bacteria after simethicone experiments. (a) Spots of *K. pneumoniae* in the pattern in which they were assessed on TSA plates. Simethicone level (mg per 100 µl) is indicated at the top. B, bacteria; D, disinfectant; N, neutralization broth; (b) Examples of spot scoring levels: −, no growth; +, 1–10 colonies; ++, 11–50 colonies; +++, >50 colonies and ++++, lawn.

Results were recorded to reflect the robustness of bacterial growth on the TSA plates following the incubation. The absence of growth in these assays is indicated by “−” and robust growth (a lawn on the spotted surface) is indicated by “++++”. Intermediate levels of growth are indicated by “+” for 1–10 colonies, “++” for 11–50 colonies and “+++” for >50 colonies but less than a lawn. Representative images of a spotted plate with lawns and individual spots assessed at each “+” indicator are shown in Fig. 1. A minimum of five independent experiments were performed for each test organism. Each independent experiment was carried out by three individuals on different weeks using different batches of prepared media and neutralizing broth to confirm the reproducibility and repeatability of the designed experiment under the conditions tested. Two individuals performed the experiment two times each, on different weeks, and one individual performed this experiment one time. The presented data represent the number of “+” for each well in a set. Occasionally, one or two wells of a replicate set showed a deviation from the listed “+” value, as indicated by the footnotes. In instances where the final c.f.u. was tested at time points, bacteria were taken from a TSB-only well, serially diluted in PBS and plated on TSA plates for colony count. All procedures were carried out in a biological safety cabinet.

### Blood control assay

The blood control assay was performed in a manner similar to that of the simethicone microtitre assay. Bacterial inoculum was prepared exactly in the same manner. For plate set-up, 198 µl sheep blood (Lampire Biological Laboratories Inc., Pipersville, PA, USA) was added to the entire first column of the 96-well plate. Next, 99 µl of the blood from these wells was serially diluted (1 : 1) into TSB ten times across the plate using a multichannel pipettor, removing and discarding 99 µl from the last set of wells so that each well had 99 µl. A volume of 99-µl TSB was added to the last column (no blood) for controls. One microlitre of the inoculum (10^9^ c.f.u. ml^−1^) was then added to each well, resulting in a final bacterial level of 10^6^ c.f.u. per well. Plates were then incubated statically at 37 °C for 4±2 and 24±2 h. At each time point, one row (11 blood dilutions and TSB alone) received 50 µl disinfectant and then 50 µl neutralizing broth (added sequentially and incubated as described earlier for the simethicone microtitre assay), one row received disinfectant alone, one row received neutralizing broth alone and one row received only PBS; 50 µl PBS replaced disinfectant and/or neutralizing broth in the wells that did not receive these. Next, 10 µl from each tested well were spotted onto a TSA plate, and the plate was incubated at 37 °C overnight. Growth was assessed as described earlier for the simethicone microtitre assay. Three independent experiments were performed, and the data represent the number of “+” for each well in a set. In instances where the final c.f.u. was tested at time points, bacteria were taken from a TSB-only well, serially diluted in PBS and plated on TSA plates for colony count. All procedures were carried out in a biological safety cabinet.

### Impact of simethicone on bacterial viability

A test was performed to assess whether simethicone alone affects bacterial viability. One microlitre bacterial inoculum, prepared as described earlier, was added to 99 µl (66 mg ml^−1^) simethicone in a 96-well plate. The plate was then incubated for 4 h at 37 °C. After incubation, 100 µl PBS was added to each well, and the entire contents were collected and plated on a single TSA plate for each well; PBS was added to thin out the simethicone so that it can be collected. As a control for low nutrient conditions present due to the lack of TSB used in this test set-up, duplicate sets of wells of bacteria were similarly inoculated into PBS. Three independent experiments were performed.

## Results

### Simethicone does not affect the efficacy of Cidex OPA against *E. coli*

To test whether simethicone affects the bactericidal efficacy of Cidex OPA disinfectant, the *E. coli* strain ATCC 35378 was incubated with varying concentrations of commercially available simethicone, and growth was assessed after 4, 18 and 24 h (±2 h). As described in Methods, this assay tested 0, 8.2, 16, 33 and 66 mg ml^−1^ simethicone, with 66 mg ml^−1^ being the highest achievable concentration (i.e. directly added from the commercial solution without dilution). While these simethicone concentrations are higher than the current clinical recommendations as described earlier (≤0.5%), these higher simethicone concentrations (8.2–66 mg ml^−1^ or 0.82–6.6%) were tested as worst-case use scenarios. Essentially, no bacterial growth was observed when disinfectant was added, whereas, in the absence of disinfectant, a bacterial lawn was visible (Table 1). These effects were independent of the presence of neutralizing broth. Regarding the disinfectant/neutralizing broth wells, it is important to note that the neutralizing broth was added after the disinfectant step. Thus, the lack of bacteria in these wells indicates that the contact time between bacteria and disinfectant was sufficient to kill or irreversibly inactivate the bacteria before the neutralizing broth was added. Occasionally, at lower simethicone concentrations, a few colonies were apparent, although these instances were rare (see Fig. 1 for examples). Notably, although the initial bacterial inoculum was 10^6^ c.f.u., by the 4 h time point, the c.f.u. had increased to 10^7^. The c.f.u. continued to increase over time, reaching 10^9^ and 10^8^ by the 18 and 24 h time points, respectively. Thus, the microbial challenge to disinfectant was higher by the later time points. Additionally, no bacterial growth was evident in the 66 mg ml^−1^ wells at all time points, even in the absence of a disinfectant (Table 1).

**Table 1.** *E. coli* growth after disinfectant treatment

	Simethicone (mg ml^−1^)			66	33	16	8.2	0
D	N	Time point (h)	Time point c.f.u.	Growth rating				
✓	✓	4	10^7^	−	−	−	−	−
		18	10^9^	−	−	−	−	−
		24	10^8^	−	−	−	−	−
✓	X	4	10^7^	−	−	−	−	−
		18	10^9^	−	−	−	−	−
		24	10^8^	−	−	−	−	−
X	✓	4	10^7^	−	++++	++++	++++	++++
		18	10^9^	−	++++	++++	++++	++++
		24	10^8^	−	++++	++++	++++	++++
X	X	4	10^7^	−	++++	++++	++++	++++
		18	10^9^	−	++++	++++	++++	++++
		24	10^8^	−	++++	++++	++++	++++

D: disinfectant (✓, present; and X, absent).

N: neutralizer (✓, present; X, absent).

Time point: the time after incubation at which a disinfectant was added.

Time point c.f.u.: control c.f.u. achieved after incubation for the specified time.

Growth rating: −, no growth; +, 1–10 colonies; ++, 11–50 colonies; +++, >50 colonies and ++++, lawn.

Initial inoculum per well: 106 c.f.u.

* 1/5 replicates was rated as ++.

† 1/5 replicates was rated as +.

### The microtitre assay demonstrated blood interference with the disinfectant activity of Cidex OPA against *E. coli*

Because no growth was observed in nearly every well with simethicone and disinfectant, a control was developed to confirm that the microtitre assay can successfully detect inhibition of disinfectant bactericidal efficacy. Blood is known to interfere with disinfectant efficacy [19]. Thus, blood was substituted for simethicone, and *E. coli* growth was tested at 4 and 24 h. In contrast to the results with simethicone, high levels of *E. coli* were present after disinfectant treatment (Table 2), demonstrating that the blood had interfered with disinfectant efficacy. The impact that the blood had on disinfection varied based on the concentration used. At the 4 h time point, there was a small amount of growth evident in 100% blood wells after disinfectant treatment, and this growth increased with decreasing blood concentration, reaching a peak at 12.5–25% blood and then decreasing. No growth was apparent in ≤3.125% blood wells at 4 h after disinfectant treatment. At the 24 h time point with disinfectant, the highest level of growth was observed in every well until 0.1% blood; no growth was evident in 0% blood. Without disinfectant, lawns of growth were observed from all wells, independent of the time point. Wells were inoculated with 10^6^ c.f.u. *E. coli* and reached 10^7^ c.f.u. by 4 h and 10^8^–10^9^ c.f.u. by 18–24 h. This result confirmed that the microtitre assay is able to demonstrate when a substance causes interference with bactericidal efficacy.

**Table 2.** *E. coli* growth in blood after disinfectant treatment

Blood (mg ml^−1^)				100%	50%	25%	12.5%	6.25%	3.125%
D	N	Time point (h)	Time point c.f.u.	Growth rating					
✓	✓	4	10^7^	+	++	++++	++++	++	−
		24	10^8^	++++	++++	++++	++++	++++	++++
✓	X	4	10^7^	+	++	++++	++++	+	−
		24	10^8^	++++	++++	++++	++++	++++	++++
X	✓	4	10^7^	+	++++	++++	++++	++++	++++
		24	10^8^	++++	++++	++++	++++	++++	++++
X	X	4	10^7^	++	++++	++++	++++	++++	++++
		24	10^8^	++++	++++	++++	++++	++++	++++
**Blood (mg ml^−1^)**				**1.56%**	**0.78%**	**0.39%**	**0.2%**	**0.1%**	**0%**
**D**	**N**	**Time point (h)**	**Time point c.f.u.**	**Growth rating**					
✓	✓	4	10^7^	−	−	−	−	−	−
		24	10^8^	++++	++++	++++	++++	+++	−
✓	X	4	10^7^	−	−	−	−	−	−
		24	10^8^	++++	++++	++++	++++	+++	−
X	✓	4	10^7^	++++	++++	++++	++++	++++	++++
		24	10^8^	++++	++++	++++	++++	++++	++++
X	X	4	10^7^	++++	++++	++++	++++	++++	++++
		24	10^8^	++++	++++	++++	++++	++++	++++

D: disinfectant (✓, present; X, absent).

N: neutralizer (✓, present; X, absent).

Time point: the time after incubation at which a disinfectant was added.

Time point c.f.u.: control c.f.u. achieved after incubation for the specified time.

Growth rating: −, no growth; +, 1–10 colonies; ++, 11–50 colonies; +++, >50 colonies and ++++ lawn.

Inoculum per well: 106 c.f.u.

### Simethicone does not affect the efficacy of Cidex OPA against *K. pneumoniae*

Testing was performed to determine whether simethicone affects the antimicrobial efficacy of Cidex OPA against *K. pneumoniae*. As with *E. coli*, essentially, no bacterial growth was observed in the presence of simethicone when disinfectant was added, although occasional colonies were observed from wells of lower concentrations of simethicone treated with disinfectant (Table 3). Lawns were evident in wells without disinfectant. Colonies were not observed in 66 mg ml^−1^ simethicone wells. Wells were initially inoculated with 10^6^ c.f.u*. K*. *pneumoniae* and continued to grow during the assay, reaching 10^7^ c.f.u. by 4 h and 10^8^ c.f.u. by 18 and 24 h.

**Table 3.** *K. pneumoniae* growth after disinfectant treatment

	Simethicone (mg ml^−1^)			66	33	16	8.2	0
D	N	Time point (h)	Time point c.f.u.	Growth rating				
✓	✓	4	10^7^	−	−	−*	−	+/−†
		18	10^8^	−	−	−	−	+‡
		24	10^8^	−	−	−	−	−§
✓	X	4	10^7^	−	−	−	−	−
		18	10^8^	−	−	−	−	−
		24	10^8^	−	−	−	−	−
X	✓	4	10^7^	−	++++	++++	++++	++++
		18	10^8^	−	++++	++++	++++	++++
		24	10^8^	−	++++	++++	++++	++++
X	X	4	10^7^	−	++++	++++	++++	++++
		18	10^8^	−	++++	++++	++++	++++
		24	10^8^	−	++++	++++	++++	++++

D: disinfectant (✓, present; X, absent).

N: neutralizer (✓, present; X, absent).

Time point: the time after incubation at which a disinfectant was added.

Time point c.f.u.: control c.f.u. achieved after incubation for the specified time.

Growth rating: −, no growth; +, 1–10 colonies; ++, 11–50 colonies; +++, >50 colonies and ++++, lawn.

Initial inoculum per well: 106 c.f.u.

* 1/5 replicates was rated as +.

† 1/5 replicates was rated as +++.

‡ 1/5 replicates was rated as ++++.

§ 1/5 replicates was rated as ++.

### Simethicone does not affect the efficacy of Cidex OPA against *S. aureus*

Table 4 shows the results of testing with *S. aureus*. Only one well out of five experiments showed any growth after disinfectant treatment. Lawns were evident in wells without disinfectant. Colonies were only observed in one 66 mg ml^−1^ simethicone well, which was not treated with a disinfectant. Wells were inoculated with 10^6^ c.f.u. *S*. *aureus* and reached 10^7^–10^8^ c.f.u. by 4 h and 10^8^–10^9^ c.f.u. by 18–24 h.

**Table 4.** *S. aureus* growth after disinfectant treatment

	Simethicone (mg ml^−1^)			66	33	16	8.2	0
D	N	Time point (h)	Time point c.f.u.	Growth rating				
✓	✓	4	10^7^–10^8^	−	−	−	−	−
		18	10^8^–10^9^	−	−	−	−†	−
		24	10^8^–10^9^	−	−	−	−	−
✓	X	4	10^7^–10^8^	−	−	−	−	−
		18	10^8^–10^9^	−	−	−	−	−
		24	10^8^–10^9^	−	−	−	−	−
X	✓	4	10^7^–10^8^	−*	++++	++++	++++	++++
		18	10^8^–10^9^	−	++++	++++	++++	++++
		24	10^8^–10^9^	−	++++	++++	++++	++++
X	X	4	10^7^–10^8^	−	++++	++++	++++	++++
		18	10^8^–10^9^	−	++++	++++	++++	++++
		24	10^8^–10^9^	−	++++	++++	++++	++++

D: disinfectant (✓, present; X, absent).

N: neutralizer (✓, present; X, absent).

Time point: the time after incubation at which a disinfectant was added.

Time point c.f.u.: control c.f.u. achieved after incubation for the specified time.

Growth rating: −, no growth; +, 1–10 colonies; ++, 11–50 colonies; +++, >50 colonies and ++++, lawn.

Initial inoculum per well: 106 c.f.u.

* 1/5 replicates was rated as +.

† 1/5 replicates was rated as ++.

### Simethicone does not affect the efficacy of Cidex OPA against *P. aeruginosa*

Finally, testing was performed to investigate whether simethicone affects the antimicrobial efficacy of Cidex OPA against *P. aeruginosa*. Unexpectedly, at the 4 h time point, a few wells displayed light-to-heavy *P. aeruginosa* growth after disinfectant treatment (Table 5). At the 18 and 24 h time points, nearly every well displayed some bacterial growth after disinfectant treatment, even in the absence of simethicone (0 mg ml^−1^). These results indicate a much higher growth in the presence of simethicone and disinfectant than with the other species tested (i.e. a decreased bactericidal efficacy), although some inhibition was observed. In the absence of a disinfectant, heavy growth was observed in 0–33 mg ml^−1^ simethicone wells, and moderate growth was observed in 66 mg ml^−1^ simethicone wells (Table 5). Wells were inoculated with 10^6^ c.f.u. *P*. *aeruginosa* and reached 10^7^ c.f.u. by the 4 h time point and 10^8^–10^9^ c.f.u. by 18–24 h.

**Table 5.** *P. aeruginosa* growth after disinfectant treatment

	Simethicone (mg ml^−1^)			66	33	16	8.2	0
D	N	Time point (h)	Time point c.f.u.	Growth rating				
✓	✓	4	10^7^	−	−	+*	+*	+/−†
		18	10^8^–10^9^	+	+	+++	++	++
		24	10^8^–10^9^	+	++	++	++	+
✓	X	4	10^7^	−	−	+*	+*	−‡
		18	10^8^–10^9^	+	+	+++	+++	++
		24	10^8^–10^9^	+	+	+++	++	+
X	✓	4	10^7^	++§	++++	++++	++++	++++
		18	10^8^–10^9^	++	++++	++++	++++	++++
		24	10^8^–10^9^	+++	++++	++++	++++	++++
X	X	4	10^7^	++§	++++	++++	++++	++++
		18	10^8^–10^9^	++	++++	++++	++++	++++
		24	10^8^–10^9^	++	++++	++++	++++	++++

D: disinfectant (✓, present; X, absent).

N: neutralizer (✓, present; X, absent).

Time point: the time after incubation at which a disinfectant was added.

Time point c.f.u.: control c.f.u. achieved after incubation for the specified time.

Growth rating: −, no growth; +, 1–10 colonies; ++, 11–50 colonies; +++, >50 colonies and ++++, lawn.

Initial inoculum per well: 106 c.f.u.

* 1/5 replicates was rated as ++++.

† 1/5 replicates was rated as +++.

‡ 1/5 replicates was rated as +.

§ 2/5 replicates were rated as ++++.

To understand the discrepancy between *P. aeruginosa* results and the results of the other three bacteria in the presence of simethicone and disinfectant, the actual time points at which testing occurred were examined. That is, the test procedures stipulated 4±2 h for the first time point; individual replicate experiments represented in Table 5 were actually assayed at 2.5, 3.25 and 4 h. Due to the rapid growth of *P. aeruginosa*, and its natural resistance to antimicrobials and biocides [20], it was reasoned that as time progressed, the c.f.u. in the wells quickly reached a point beyond the rated antimicrobial activity of Cidex OPA. Thus, the 4 h time point data from Table 5 was split into the actual time points tested, additional experiments were performed to reach five replicates at each time point, and a 1 h time point was included. As shown in Table 6, when c.f.u. in the well remains at 10^6^ c.f.u., no bacterial growth is evident. However, by 3.25–4 h, the c.f.u. increased to 10^7^, and occasional bacterial colonies were observed.

**Table 6.** *P. aeruginosa* growth after disinfectant treatment in early time points

	Simethicone (mg ml^−1^)			66	33	16	8.2	0
D	N	Time point (h)	Time point c.f.u.	Growth rating				
✓	✓	1	10^6^	−	−	−	−	−
		2.5	10^6^	−	−	−	−	−
		3.25	10^7^	−	−	−	−	−
		4	10^7^	−	−	+*	+*	+/−†
✓	X	1	10^6^	−	−	−	−	−
		2.5	10^6^	−	−	−	−	−
		3.25	10^7^	−	−	−	−	−
		4	10^7^	−	−	+*	++	++
X	✓	1	10^6^	−	++++	++++	++++	++++
		2.5	10^6^	−	++++	++++	++++	++++
		3.25	10^7^	−	++++	++++	++++	++++
		4	10^7^	−	++++	++++	++++	++++
X	X	1	10^6^	−	++++	++++	++++	++++
		2.5	10^6^	−	++++	++++	++++	++++
		3.25	10^7^	−	++++	++++	++++	++++
		4	10^7^	−	++++	++++	++++	++++

D: disinfectant (✓, present; X, absent).

N: neutralizer (✓, present; X, absent).

Time point: the time after incubation at which a disinfectant was added.

Time point c.f.u.: control c.f.u. achieved after incubation for the specified time.

Growth rating: −, no growth; +, 1–10 colonies; ++, 11–50 colonies; +++, >50 colonies and ++++, lawn.

Initial inoculum per well: 106 c.f.u.

* 1/3 replicates was rated as ++++.

† 1/3 replicates was rated as +++.

### Simethicone exhibits bactericidal activity at high concentrations

As mentioned earlier, there was generally no bacterial growth observed in wells with 66 mg ml^−1^ simethicone, even in the absence of a disinfectant. It was initially hypothesized that this effect may be influenced by a lack of available nutrients or direct microbial killing by the simethicone solution or both. Of note, there was reduced growth in the 100% blood wells (Table 2). To distinguish between these possibilities, growth was monitored after incubation with 66 mg ml^−1^ simethicone or PBS for 4 h. Although 4 h incubation in PBS resulted in lawns of bacteria after plating, zero bacterial colonies were observed from wells of bacteria incubated with 66 mg ml^−1^ simethicone for 4 h (Table 7). These data indicate a strong impact on microbial viability by 66 mg ml^−1^ simethicone through a mechanism likely to be unrelated to nutrient starvation.

**Table 7.** Impact of 66 mg ml^−1^ simethicone on microbial viability

	4 h Treatment	
Species	Simethicone (c.f.u.)	PBS
*E. coli*	0	Lawn
*P. aeruginosa*	0	Lawn
*K. pneumoniae*	0	Lawn
*S. aureus*	0*	Lawn

Inoculum per well: 106 c.f.u.

* 1/7 replicate samples had one colony present.

## Discussion

While simethicone use during gastrointestinal endoscopy can enhance visualization during those procedures, it has been unclear whether residual simethicone reduces the bactericidal efficacy of disinfectant, either through masking of microbial soil or through other physical/chemical interactions. The results presented here demonstrate that, under the conditions tested, Cidex OPA retained strong bactericidal efficacy in the presence of simethicone, particularly when the bacterial c.f.u. remained at 10^6^–10^7^ c.f.u. (Tables1 and 3^6^, Fig. 1), although one limitation of this approach is the use of the semiquantitative scale versus c.f.u. enumeration. Notably, this result was observed for each of the four different bacterial species, including three Gram-negative (*E. coli*, *K. pneumoniae* and *P. aeruginosa*) microorganisms and one Gram-positive (*S. aureus*) microorganism. These species are among the most associated with gastrointestinal endoscope contamination [4,16, 18, 21,23]. A noted caveat to these results is that simethicone is insoluble in water, so the actual concentrations of the simethicone in the wells cannot be confirmed; this is a limitation of this study. Notably, however, simethicone/TSB emulsions were mixed five to ten times between dilutions, so the listed simethicone concentrations are likely accurate. Importantly, substituting blood for simethicone did interfere with disinfectant bactericidal activity (Table 2), indicating that the microtitre assay used can demonstrate interference with bactericidal efficacy, in the presence of an interfering substance. This important control also underscores how critical it is to remove blood from reusable medical devices, as even low concentrations of blood can inhibit disinfectant efficacy (Table 2).

Not surprisingly, a decrease in disinfectant efficacy was observed as the starting inoculum of 10^6^ c.f.u. increased over time. Disinfection processes are generally expected to achieve a minimum 6-log reduction of vegetative test microorganisms [24]. Similarly, US Food and Drug Administration (FDA)-cleared high-level disinfectants [25] are recommended to demonstrate efficacy against vegetative bacteria following the AOAC International Use-Dilution Methods [26]. Therefore, it was expected that a 10^6^ c.f.u. inoculum may result in <1 c.f.u., and “−” results in plate spots. However, in the experiments presented herein, bacteria were incubated with simethicone and TSB at 37 °C for 4, 18 or 24 h before the addition of disinfectant, so c.f.u. increased beyond the initial 10^6^ c.f.u. inoculum size, and some bacteria remained after disinfectant treatment, resulting in occasional colonies on the spot plates. This effect was particularly evident with *P. aeruginosa* (Table 5). In support of these *P. aeruginosa* findings, it was previously discovered that a large percentage of *P. aeruginosa* clinical isolates, particularly antibiotic-resistant strains, showed decreased susceptibility to OPA solutions at 10^8^ c.f.u. ml^−1^ [20]. However, for all species tested here, when c.f.u. remained at 10^6^, the disinfectant was highly effective in the presence of simethicone (Table 6). This phenomenon might also explain why occasional colonies appeared in disinfectant-treated wells of *E. coli*, *K. pneumoniae* and *S. aureus*, especially, at later time points; even after 4 h of incubation, c.f.u. reached 10^7^ for each species (Tables1^4^). In the case of *K. pneumoniae* and *P. aeruginosa*, individual wells occasionally presented less than 6-log reduction (see Table 3, footnote †, and Table 5, footnotes ∗ and †), and we speculate that this can in part be due to a difference in OPA concentration than is recommended by the manufacturer, differences between this method and the Use Dilution Method (e.g. presence of TSB in this method) and/or biofilm formation during the incubation periods. It is important to note, however, that in all cases where c.f.u. at the time of treatment was 10^6^, <1 colony appeared, indicating a 6-log unit reduction. The disinfectant used generally exhibited greater than this effect in these studies.

Surprisingly, and in contrast to initial concerns about the impact on disinfectant activity, the higher test concentration of simethicone exhibited bactericidal activity (Table 7). After a 4 h incubation with the highest achievable simethicone concentration (66 mg ml^−1^), essentially all bacteria in the well were non-viable, and this effect appears to be unrelated to nutrient limitation. We speculate that this effect results from a physical or chemical interaction between the simethicone, or one of the components in the commercial simethicone preparation, and the bacteria. Of note, the commercial simethicone formulation used in this study contains known preservatives, including polysorbate 60 and sodium benzoate, and these preservatives can explain, either in part or in full, the bactericidal activity of simethicone at the highest concentration (66 mg ml^−1^). Because the lower concentrations of simethicone (even 50%) did not seem to exhibit this activity, it appears that there is a critical simethicone (or simethicone/preservative mixture) concentration needed to achieve this result. As described earlier, 66 mg ml^−1^ is approximately 13 times higher than the current highest recommended clinical use concentration, so the concentration of preservatives in current clinical uses is likely very small. Future studies are needed to elucidate this issue further.

It is important to note that the purpose of the study was not to demonstrate the efficacy of Cidex OPA as a high-level disinfectant but to investigate whether simethicone reduced the bactericidal effects on the test organisms. Overall, the data indicate that simethicone does not reduce the bactericidal efficacy of the disinfectant, under the conditions tested. Limitations of this study include: the method does not assess the impact that cleaning may have to physically remove simethicone prior to exposure to the disinfectant, only a select set of microorganisms were tested under *in vitro* conditions, it does not address whether residual simethicone can influence subsequent biofilm formation of microorganisms, it does not investigate potential infections as a result of retained microorganisms, it does not utilize medical devices as the inoculated surface or otherwise replicate simulated use testing and it only assesses the impact of simethicone on a single high-level disinfectant. It will be important in future experiments to test additional clinically relevant bacteria and other disinfectants.

Additional studies might also include designs that more closely model the endoscope environment and involve biofilm-state bacteria. Biofilm-state bacteria might provide a greater challenge to disinfection than the bacterial suspensions investigated herein. Simethicone can form water-insoluble globules on medical devices, which can further complicate the disinfection of biofilms present. Further studies are also needed to evaluate simethicone effects on non-microbial soils, including any potential impacts on cleaning efficacy. With additional investigations elucidating the effect of simethicone on device soil, the impact of simethicone used for endoscopic procedures can be assessed from an infection control perspective.

## Abbreviations

HLD: high-level disinfection
OPA: ortho-phthalaldehyde
TSA: tryptic soy agar
TSB: tryptic soy broth

## References

[1] Ofstead CL, Wetzler HP, Heymann OL, Johnson EA, Eiland JE (2017). Longitudinal assessment of reprocessing effectiveness for colonoscopes and gastroscopes: results of visual inspections, biochemical markers, and microbial cultures. Am J Infect Control.

[2] Pajkos A, Vickery K, Cossart Y (2004). Is biofilm accumulation on endoscope tubing a contributor to the failure of cleaning and decontamination?. J Hosp Infect.

[3] Tian H, Sun J, Guo S, Zhu X, Feng H (2021). The effectiveness of drying on residual droplets, microorganisms, and biofilms in gastrointestinal endoscope reprocessing: a systematic review. Gastroenterol Res Pract.

[4] Alfa MJ, Singh H (2020). Impact of wet storage and other factors on biofilm formation and contamination of patient-ready endoscopes: a narrative review. Gastrointest Endosc.

[5] USFDA (2015). Reprocessing medical devices in health care settings: validation methods and labeling; guidance for industry and food and drug administration staff.

[6] ANSI/AAMI (2021). ST91:2021 flexible and semi-rigid endoscope processing in health care facilities.

[7] ANSI/AAMI (2022). ST98:2022 cleaning validation of health care products--requirements for development and validation of a cleaning process for medical devices.

[8] Lawrence CB, EH S (1968). Disinfection, Sterilization, and Preservation.

[9] Rutala WA WD (2008). Healthcare infection control practices advisory committee (HICPAC). Guideline for disinfection and sterilization in healthcare facilities. US Centers for Disease Control and Prevention.

[10] Speer T, Vickery K, Alfa M, Sáenz R (2023). Minimizing the risks of simethicone in endoscope reprocessing. J Clin Gastroenterol.

[11] Barakat MT, Huang RJ, Banerjee S (2019). Simethicone is retained in endoscopes despite reprocessing: impact of its use on working channel fluid retention and adenosine triphosphate bioluminescence values (with video). Gastrointest Endosc.

[12] Ofstead CL, Hopkins KM, Eiland JE, Wetzler HP (2019). Widespread clinical use of simethicone, insoluble lubricants, and tissue glue during endoscopy: a call to action for infection preventionists. Am J Infect Control.

[13] Ofstead CL, Wetzler HP, Johnson EA, Heymann OL, Maust TJ (2016). Simethicone residue remains inside gastrointestinal endoscopes despite reprocessing. Am J Infect Control.

[14] Devereaux BM, Taylor ACF, Athan E, Wallis DJ, Brown RR (2019). Simethicone use during gastrointestinal endoscopy: position statement of the Gastroenterological Society of Australia. J Gastroenterol Hepatol.

[15] Day LW, Muthusamy VR, Collins J, Kushnir VM, Sawhney MS (2021). Multisociety guideline on reprocessing flexible GI endoscopes and accessories. Gastrointest Endosc.

[16] Balan GG, Sfarti CV, Chiriac SA, Stanciu C, Trifan A (2019). Duodenoscope-associated infections: a review. Eur J Clin Microbiol Infect Dis.

[17] Kovaleva J (2017). Endoscope drying and its pitfalls. J Hosp Infect.

[18] Mehta AC, Muscarella LF (2020). Bronchoscope-related “superbug” infections. Chest.

[19] CDC (2008). Factors affecting the efficacy of disinfection and sterilization. Guideline for disinfection and sterilization in healthcare facilities: US Centers for Disease Control and Prevention.

[20] Herruzo R, Vizcaíno MJ, Herruzo I (2017). An exception to the rule “no association between antibiotic resistance and decreased disinfectant microbicidal efficacy”: Orthophthalaldehyde (OPA) and *Pseudomonas aeruginosa* isolated from ICU and paraplegic patients. J Prev Med Hyg.

[21] Kovaleva J, Peters FTM, van der Mei HC, Degener JE (2013). Transmission of infection by flexible gastrointestinal endoscopy and bronchoscopy. Clin Microbiol Rev.

[22] Houri H, Aghdaei HA, Firuzabadi S, Khorsand B, Soltanpoor F (2022). High prevalence rate of microbial contamination in patient-ready gastrointestinal endoscopes in Tehran, Iran: an alarming sign for the occurrence of severe outbreaks. Microbiol Spectr.

[23] USFDA Use duodenoscopes with innovative designs to enhance safety: FDA safety communication. https://www.fda.gov/medical-devices/safety-communications/use-duodenoscopes-innovative-designs-enhance-safety-fda-safety-communication.

[24] AAMI (2023). TIR12:2020/(R)2023 designing, testing, and labeling medical devices intended for processing by health care facilities: a guide for device manufacturers.

[25] USFDA (2000). Content and format of premarket notification [510(k)] submissions for liquid chemical sterilants/high level disinfectants.

[26] AOAC Inc (1990). Official methods of analysis of the association of the official analytical chemists.

